# Corruption, inequality and population perception of healthcare quality in Europe

**DOI:** 10.1186/1472-6963-13-472

**Published:** 2013-11-11

**Authors:** Zlatko Nikoloski, Elias Mossialos

**Affiliations:** 1 London School of Economics, Houghton Street, WC2A 2AE London, UK

**Keywords:** Quality of healthcare, Access to healthcare, Corruption, Inequality, EU

## Abstract

**Background:**

Evaluating the quality of healthcare and patient safety using general population questionnaires is important from research and policy perspective. Using a special wave of the Eurobarometer survey, we analysed the general population’s perception of health care quality and patient safety in a cross-country setting.

**Methods:**

We used ordered probit, ordinary least squares and probit analysis to estimate the determinants of health care quality, and ordered logit analysis to analyse the likelihood of being harmed by a specific medical procedure. The models used population weights as well as country-clustered standard errors.

**Results:**

We found robust evidence for the impact of socio-demographic variables on the perception of quality of health care. More specifically, we found a non-linear impact of age on the perception of quality of health care and patient safety, as well as a negative impact of poverty on both perception of quality and patient safety. We also found robust evidence that countries with higher corruption levels were associated with worse perceptions of quality of health care. Finally, we found evidence that income inequality affects patients’ perception vis-à-vis safety, thus feeding into the poverty/health care quality nexus.

**Conclusions:**

Socio-demographic factors and two macro variables (corruption and income inequality) explain the perception of quality of health care and likelihood of being harmed by adverse events. The results carry significant policy weight and could explain why targeting only the health care sector (without an overall reform of the public sector) could potentially be challenging.

## Background

The health care systems of the European Union (EU) member states have been subject to continuous reform over the last twenty years, mainly stemming from the pressures of aging populations and challenges in reforming public budgets. Over the same period, the EU witnessed its biggest enlargement (the so called *big bang*) that brought in ten new countries – almost all of which belong to the group of ‘transition’ countries. The enlargement in 2004 (and the subsequent one in 2007)^a^ also allowed for the possibility of both patients and medical staff to migrate across borders, thus adding further layers of complexity to the extant national health care systems. Given this background, an evaluation of people’s perception of health care quality (and of patient safety) is a high-priority task, not least because a careful examination of factors that influence perceptions of health care quality could provide the basis for effective policy action aimed at improving access to services and the quality of national health care. Having said that, evaluating the quality of health care is not a new topic. Indeed, to date, there have been many systematic cross-country reviews (and subsequent ranking) of health care systems. Some of them have tried to understand the quality of national health care from a comparative perspective, while others (relying on surveys of patient satisfaction/perception of quality) help to provide a deeper understanding of the determinants of health care quality at national level.

Using the Eurobarometer 327 (2009) survey, we look at the general population’s perception of health care quality and patient safety in a cross-country EU setting. This paper adds to the extant literature in three crucial ways: (i) we study the macro-level and socio-demographic determinants of the general population’s perception of the quality of healthcare; (ii) building on (i), we disentangle which aspects of health care are the most important in shaping the perception of health care quality; and (iii) we analytically explore the macro-level and socio-demographic determinants of the likelihood of being harmed by medical procedures. While doing so, we employ additional robustness checks. It is important to note that there are two limitations to our findings: the lack of control variables vis-à-vis personal-level health care utilisation rates and subjective health evaluations. We have addressed these shortcomings by using macro-proxy variables for health care utilisation (health care expenditure as a percentage of gross domestic product (GDP)) as well as controlling for age, which is usually strongly correlated with subjective health status evaluation.

A substantial literature focuses on the issue of quality of health care (both qualitative and quantitative). The existing qualitative body of research can be divided into two main groups, one relying on medical professionals’ perceptions of quality (see, for example, Robinson et al [1]) and the other solely focusing on patients^b^. Patient surveys could offer a better overview of perception of health care quality and safety [2]. According to the authors, evidence shows that patient perceptions are distinctive, and often conflicting, compared with those of the health care providers, which ultimately leads to different assessments of health care quality. Given that patients are the final users of health care services, considering their perceptions would be more important. Furthermore, most of the studies using patient surveys are small-scale studies that use patient interviews and focus groups (Anderson et al [3], Attree [4], Concato and Feinstein [5], Gerteis et al [6], Irurita [7], Jun et al [8], Larrabee and Bolden [9], Ngo-Metzger et al [10], Radwin [11], Stichler and Weiss [12], and Ware and Stewart [13]). Finally, within the body of research that focuses on patients, a sub-section concentrates on patient satisfaction with health care services as the only measure of health care quality (Taylor and Cronin [14], Babakus and Mangold [15], Sohail [16]). However, this subsection of literature has been fairly controversial and has received significant criticism from the research community (for a detailed discussion , see Crow et al [17]).

Another strand of literature has looked at perceptions of quality of health care from a quantitative and cross-country perspective. To date, there have been two papers that have looked at the issue of patient quality from this perspective. Cleary et al [18] analysed the quality of health care using patient-reported measures of quality in the United States, Australia, Canada, New Zealand and the United Kingdom. Wendt et al [19] used a truncated sample of 14 EU countries to assess the preferences for state involvement, and subsequently, quality of health care across the EU.

In addition, a significant body of literature has looked at the determinants of what constitutes the core attributes of health service quality. Gronroos [20] argues that these attributes can be divided into two groups: functional (ambience, i.e. description of the form in which the service is delivered) and technical (outcome, i.e. the quality of what is delivered). Similar reasoning is found in other studies on the topic (Zifko-Baliga and Krampf [21], DeRuyter and Wetzels [22]).

To the best of our knowledge, there is one study concerning patient safety in a cross-country setting, assessing the likelihood of medication and medical errors in five OECD countries (Cleary et al [18]). Finally, in the context of the US, a burgeoning literature has looked at various aspects of quality and performance of HMOs (Health Maintenance Organisation). The overall finding of that sizeable literature points to a negative link between age and perception of quality as well as a negative link between chronic illness and perception of quality (Miller and Luft [23,24],).

Following the strands of literature outlined above, in this paper we answer the following three questions:

(i) What individual and macro-level variables drive the perception of quality of healthcare among EU nationals?;

(ii) What attributes of healthcare play the biggest role when evaluating the quality of healthcare?;

(iii) What individual and macro-level variables drive the perception of the likelihood of being harmed by hospital or non-hospital care?

## Methods

### Data

The empirical analysis in this paper is based on data from the special Eurobarometer Survey 327, which is part of a set of surveys conducted on behalf of the European Commission. This special Eurobarometer survey was conducted during September and October 2009 and features attitudes towards quality of health care as well as the likelihood of experiencing adverse events. Most national samples were drawn with a multi-stage, random probability sampling design from the population aged 15 years and over. In each country, approximately 1000 standardised face-to-face interviews were conducted (for the smaller countries, like Luxembourg, Malta and Cyprus, the number was about 500). The German and UK samples consist of 1500 respondents. The total size of the sample was around 26,000 people. In this paper we analysed data for the 27 countries that currently make up the European Union. Data were weighted according to the national weighting procedure with sex, age, region and size of locality entering the iteration procedure. The data used in the paper are widely available.

### Dependent variables

In order to analyse respondents’ perceptions vis-à-vis the quality of health care and the likelihood of being harmed by hospital or non-hospital care, we focused on two questions. The first was: “How would you evaluate the overall quality of health care in our country? Answers were: (1) very good; (2) fairly good; (3) fairly bad; and (4) very bad”^c^. The question was used in an ordered probit and OLS analysis. As a robustness check we also constructed an alternative measure (0–1) that was subsequently used in a probit analysis. Table 1 provides a snapshot of the average values for this variable. As expected, the Nordic countries and the oldest welfare states of Western Europe tend to show perceptions of the highest levels of health care quality. Incidentally, these are the countries with the highest health expenditures both, per capita and as a percentage of GDP.

**Table 1:** Patients’ perception of quality of healthcare and selected healthcare systems variables

**Rank**	**Country**	**Eurobarometer mean score for perception of quality of healthcare**	**Health expenditure per capita (current USD) average 2008-2010**	**Total health expenditure (as % of GDP) average 2008-2010**	**Number of physicians per 1000 people, average 2008-2010**
1	Austria	1.613	5048.8	10.8	4.9
2	Belgium	1.671	4707.2	10.5	3.0
3	Sweden	1.74	4641.3	9.6	3.8
4	Netherlands	1.862	5527.0	11.3	2.9
5	Finland	1.866	4105.6	8.8	2.8
6	Denmark	1.895	6406.2	11.0	3.4
7	UK	1.905	3581.3	9.4	2.7
8	Luxembourg	1.918	8166.1	7.5	2.8
9	Malta	1.922	1653.6	8.5	3.1
10	France	1.955	4823.9	11.7	3.5
11	Germany	1.998	4703.1	11.3	3.6
12	Spain	2.064	3015.2	9.4	4.0
13	Czech Rep.	2.133	1482.8	7.6	3.6
14	Cyprus	2.217	1805.5	6.0	2.6
15	Estonia	2.265	959.5	6.2	3.4
16	Slovenia	2.299	2214.3	9.0	2.5
17	Italy	2.52	3347.6	9.3	3.9
18	Ireland	2.527	4732.1	9.2	3.2
19	Slovak Rep.	2.531	1428.3	8.6	3.0
20	Lithuania	2.653	850.5	7.1	3.6
21	Portugal	2.664	2371.7	10.6	3.9
22	Latvia	2.791	816.1	6.6	3.0
23	Poland	2.837	906.4	7.3	2.2
24	Hungary	2.92	1006.0	7.4	3.1
25	Bulgaria	2.931	475.2	7.0	3.7
26	Greece	2.974	2941.1	10.3	3.6
27	Romania	2.984	458.8	5.6	2.3

Sources: Eurobarometer special survey on health (2010), World Development Indicators. The perception of quality of healthcare variable takes values from 1 - very good to 4 - very bad.

Second, to analyse the possibility of being harmed by hospital or non-hospital care, we relied on the question: “How likely do you think it is that patients could be harmed by hospital (or non-hospital) care in our country? Answers were: (1) very likely; (2) fairly likely; (3) not very likely; (4) not at all likely”. Following established practice in analysing questions of likelihood, an ordered logit model was used^d^.

### Independent variables – individual characteristics

As in previous studies (Carlson et al [25], Cleary et al [26], Haviland et al [27], Roohan et al [28]) we use age as one of the main explanatory variables. Rather than using dummies for age categories (Wendt et al [19]), we use age and an age-squared term to test the possibility of a non-linear effect of age on health care quality perception. The Eurobarometer does not include variables on health status and thus we cannot control for it^e^. As in other studies, we also control for level of education (Roohan et al [28], Carlson et al [25]). Previous research attempts to control for respondents’ gender although the results from the extant research, as well as meta-analysis, tend to be inconclusive (Cleary et al. [18]).

In addition to the variables above, we also control for marital status, differences between rural and urban dwellers and size of households. Differences between income groups are an important determinant (Schoen et al [29]), especially in health care in the context of EU countries, which rely on high levels of redistribution. For this purpose, we constructed a category called poor (corresponding to the people who have difficulty paying bills most of the time over the last 12 months). Finally, to capture the relative standing of the people in the country, rather than using subjective income assessment, we used an asset index, constructed using factor analysis (for a detailed explanation of asset index creation and usage in survey analysis please refer to Nikoloski and Ajwad [30]).

### Independent variables – macro level variables

Even though the EU comprises countries that are at a high level of economic development, there is significant variation among countries in terms of macro (economic) developments. Hence, it is important to control for them. The level of development is captured by the log of the average GDP per capita (PPP) for the period 2008–2010. We also control for the level of economic inequality (3-year average Gini coefficient). There is also significant institutional variation among the countries. Given that all of them are, more or less, democratic, while there are significant differences in terms of bureaucratic quality, we use a three year average (2008–2010) of Transparency International’s Corruption Perception Index. Finally, we experiment with different health care variables. To capture the monetary input, we use data on total health care expenditure (per capita and as a percentage of GDP). The extant research uses the level of health care expenditure as an indication of the interventionist power of the state in the field of health care. As an indicator for real input, we included the number of physicians in relation to the population (per 1,000). Physicians are often the first point of contact for patients and make decisions affecting a major part of total health care resources. In many countries, they act as gatekeepers and are often responsible for transferring patients to specialist health care and further care givers. Additional file 1: Table A2 provides a more detailed description of the macro level independent variables.

Table 2 provides a snapshot of the basic macro-level variables used in our analysis. As mentioned previously, for the analytical purpose of our exercise we used ordered probits, OLS and probits (for perception of quality of health care) and ordered logits (for likelihood of being harmed by hospital or non-hospital care).

**Table 2:** Summary of statistics

**Variable**	**Obs**	**Mean**	**Std. Dev.**	**Min**	**Max**
GDP per capita, PPP (consant USD, 2005) average 2008-2010	26025	26525.14	10055.47	11166.64	70095.39
Gini, average 2008-2010	26025	29.80	3.86	23.30	37.10
Health care expenditure per capita, in USD, average 2008-2010	26025	2829.94	1289.29	814.67	6470.09
Health care expenditure (as % of GDP), average 2008-2010	26025	8.94	1.78	5.55	11.66
Nurses (per 1,000 people), average 2008-2010	26025	7.21	5.35	0.15	23.96
Physicians (per 1,000 people), average 2008-2010	26025	3.39	0.79	2.15	6.11
Transparency international corruption perception index	26025	6.392042	1.794522	3.666667	9.3
Unemployment (in %), average 2008-2010	26025	8.50	3.00	3.57	16.47

Source: World Development Indicators (WDI), Eurostat and Transparency International.

### Independent variables – criteria for selecting a doctor

Building on this, we then analysed the importance of different criteria when assessing the quality of healthcare. For this purpose we used the question: “Of the following criteria, which are the three most important criteria when you think of high quality healthcare in our country?: (a) proximity of hospital and doctors; (b) free choice of doctor; (c) respect of a patient’s dignity; (d) medical staff that is well trained; (e) a clean environment at the healthcare facility; (f) treatment that works; (g) free choice of hospital; (h) healthcare that keeps you safe from harm; (i) no waiting list to get seen and treated; (j) a welcoming and friendly environment and (k) modern medical equipment”.

## Results

### Perception of quality of care

The results of the ordered probit analysis are captured in Table 3. We find a non-linear relationship between age and perception of quality of health care (the inflection point of the curve occurs around the age of 40 years). Individuals living in households with a higher wealth index (and therefore enjoying a better socio-economic status) tend to be associated with better perceptions of health care quality. When it comes to gender, we find evidence that females are more negative (vis-à-vis perceptions of quality of health care) than males (although the magnitude of the coefficient is minuscule). Finally (and closely corresponding to our wealth index findings), the poor tend to have more negative perceptions of health care quality than the non-poor. Of the macro-level variables the Corruption Perception Index is the one which is the most interesting, lending evidence to the fact that in more transparent countries, the perception of the quality of health care tends to be higher^f^.

**Table 3:** Perceptions of quality of healthcare - ordered probit

	**(1)**	**(2)**	**(3)**
Married	0.0963***	−0.0435*	−0.0408
	(0.0368)	(0.0244)	(0.0300)
Divorced	0.00588	−0.0303	−0.0539
	(0.0434)	(0.0357)	(0.0478)
Widow	0.112**	−0.0475	−0.0376
	(0.0453)	(0.0360)	(0.0424)
Primary education	0.0285	0.0581	0.0828
	(0.0771)	(0.0655)	(0.0686)
Secondary education	0.124**	0.111**	0.0681
	(0.0612)	(0.0454)	(0.0621)
University education	0.0188	0.0241	0.0295
	(0.0553)	(0.489)	(0.0518)
Female	0.00935	0.0419**	0.0366**
	(0.0196)	(0.0193)	(0.0178)
Urban	0.0435	0.0121	0.0478
	(0.0411)	(0.0206)	(0.0326)
Household size	0.0346**	−0.00778	−0.00385
	(0.0154)	(0.00622)	(0.0104)
Poor	0.258***	0.188***	0.185***
	(0.0419)	(0.0441)	(0.0455)
Age	0.0170***	0.0169***	0.0172***
	(0.00363)	(0.00349)	(0.00347)
Age squared	−0.000293***	−0.000209***	−0.000218***
	(0.0000373)	(0.0000352)	(0.0000370)
Asset index	−0.325***	−0.0223	−0.0396**
	(0.0388)	(0.0146)	(0.0177)
_cut1	−0.824***	−0.453***	−5.765*
	(0.153)	(0.0863)	(3.264)
_cut2	0.785***	1.491***	−3.959
	(0.168)	(0.0822)	(3.239)
_cut3	1.793***	2.694***	−2.838
	(0.148)	(0.111)	(3.217)
Log of GDP per capita (average 2008-2010)			−0.404
			(0.301)
Total health expenditure (as % of GDP, average 2008-2010)			−0.00968
			(0.0481)
Gini, average 2008-2010			0.0277
			(0.0236)
Log of number of doctors per 1000 people (average 2008-2010)			−0.118
			(0.405)
Corruption perception index (average 2008-2010)			−0.206***
			(0.0551)
N	25661	25661	25661
Country dummies	No	yes	No
adj. R-sq			
Pseudo R-sq	0.039	0.161	0.114

Standard errors in parentheses.

=”*p < 0.1, **p < 0.05, ***p < 0.01”.

As a robustness check, we repeated the analysis using an OLS and probit methodology, respectively. Additional file 2: Table A1 presents these results, which, confirmed our main hypotheses distilled from the ordered probit analysis outlined above^g,h^.

We also conducted a secondary robustness check by including an interaction term between the age category and the proxy for corruption in order to test if the perception of quality of healthcare stems from the older generation (i.e. the one which is the heaviest user of healthcare services). While informative, the results did not yield statistically significant results^i^.

### Determinants of quality of healthcare

Building on the analysis and findings above, we proceeded with determining the statistical significance of different quality of health care determinants, while alternating between models that include individual characteristics, macro-level variables, and/or country dummies. Our results are presented in Table 4. It is important to note that the individual characteristics are binominal variables which are included as separate independent variables in the model. The robust results that emerge from this exercise lend evidence to the statistical significance of the following few determinants: choice of doctor, well-trained staff, and, in the majority of instances, proximity of doctor and modern medical equipment. The control variables (individual and macro-level variables) tend to maintain the same sign and significance as in the perception of quality exercise^j^.

**Table 4:** Determinants of quality of healthcare

	**Ordered probit**	**Ordered probit**	**Ordered probit**	**Ordered probit**	**OLS**	**OLS**	**OLS**	**OLS**
	**(1)**	**(2)**	**(3)**	**(4)**	**(5)**	**(6)**	**(7)**	**(8)**
Doctor_prox	−0.181***	−0.165***	−0.989***	−0.0370	−0.133***	−0.120***	−0.0596***	−0.0217
	(0.0627)	(0.0571)	(0.0276)	(0.0389)	(0.0451)	(0.0401)	(0.0164)	(0.0244)
Choice_doc	−0.946*	−0.960*	−0.0737**	−0.736*	−0.0723	−0.0722	−0.0466**	−0.0474*
	(0.0555)	(0.0554)	(0.0321)	(0.0416)	(0.0408)	(0.0401)	(0.0196)	(0.0266)
Patient_dig	0.0177	0.00907	0.0222	0.0667	0.00804	0.00653	0.0107	0.0422
	(0.0527)	(0.0529)	(0.0438)	(0.441)	(0.0404)	(0.0398)	(0.0270)	(0.0297)
Trained	−0.255***	−0.240***	−0.116***	−0.119***	−0.188***	−0.174***	−0.0696***	−0.0732***
	(0.0492)	(0.0479)	(0.0280)	(0.0268)	(0.0360)	(0.398)	(0.0270)	(0.0297)
Clean	−0.113	−0.131*	0.00187	0.0178	−0.0826	−0.0942	−0.000917	0.0122
	(0.787)	(0.0771)	(0.0337)	(0.0565)	(0.0585)	(0.0562)	(0.0206)	(0.0363)
Effective	−0.0431	−0.0410	−0.0602*	−0.0420	−0.0332	−0.0310	−0.0373*	−0.0258
	(0.0652)	(0.0614)	(0.0337)	(0.0444)	(0.0484)	(0.0448)	(0.0205)	(0.0287)
Hosp_choice	−0.108*	−0.101*	−0.0338	−0.0223	−0.0805*	−0.0738	−0.0223	−0.0131
	(0.0631)	(0.0614)	(0.367)	(0.0429)	(0.0468)	(0.0449)	(0.0222)	(0.0275)
No_harm	−0.0875	−0.0959	0.0153	0.0292	−0.663	−0.0710	0.00669	0.0177
	(0.697)	(0.0677)	(0.0299)	(0.0428)	(0.0513)	(0.0491)	(0.0184)	(0.0274)
No_waitlist	−0.0699	−0.0557	0.0549	0.159***	−0.0568	−0.0454	0.0280	0.0940**
	(0.0937)	(0.0886)	(0.0346)	(0.0525)	(0.0703)	(0.0653)	(0.0211)	(0.0353)
Friendly	−0.0735	−0.0711	−000400	−0.0479	−0.0541	−0.0513	−0.0233	−0.0296
	(0.0489)	(0.0499)	(0.0469)	(0.0507)	(0.0363)	(0.0363)	(0.0283)	(0.0331)
Modern_equip	−0.106*	−0.109*	−0.124***	−0.129***	−0.0794*	−0.0803*	−0.0718***	−0.0808***
	(0.0634)	(0.0620)	(0.0405)	(0.0408)	(0.0464)	(0.0446)	(0.0235)	(0.0255)
_cut1	−1.429***	−1.216***	−0.594***	−6.527***				
	(0.124)	(0.156)	(0.115)	(3.268)				
_cut2	0.107	0.347***	1.359***	−4.704				
	(0.142)	(0.171)	(0.109)	(3.245)				
_cut3	1.069***	1.327***	2.566***	−3.575				
	(0.120)	(0.154)	(0.141)	(3.227)				
_cons					2.524***	2.354***	1.816***	5.707**
					(0.0963)	(0.115)	(0.0655)	(2.059)
N	25661	25661	25661	25661	25661	25661	25661	25661
R-sq					0.013	0.042	0.318	0.243
adj. R-sq					0.013	0.041	0.317	0.242
Pseudo R-sq	0.006	0.018	0.163	0.120				
Individual socio-economic characterestics	No	Yes	Yes	Yes	No	Yes	Yes	Yes
Macro level variables	No	No	No	No	No	No	No	No
Contry dummies	No	No	Yes	No	No	No	Yes	No

Standard errors in parentheses.

=”*p < 0.1, **p < 0.05, ***p < 0.01”.

### Likelihood of experiencing an adverse event

We then proceeded with analysing the likelihood of experiencing a hospital or non-hospital adverse event. The results of our ordered logit analysis are captured in Table 5. First, the results suggest that the statistical significance of the likelihood of experiencing a non-hospital adverse event is lower compared with the results of the analysis conducted on the likelihood of experiencing a hospital-related adverse event. However, there are some interesting results associated with individual socio-demographic characteristics. As in some of the cases above, we find evidence for the existence of a non-linear relationship between age and the likelihood of experiencing an adverse event. This relationship, however, is exhibited only in the context of hospital-related adverse events (the results are insignificant in the case of non-hospital care). As in some of the exercises conducted above, here as well, the poor tend to have higher expectations for the possibility of experiencing an adverse event. Interestingly, we find evidence that in countries with higher inequality, the perceived likelihood of experiencing adverse events among respondents tends to be higher. In a way, this finding is a variation of the poor people-likelihood of experiencing hospital/non-hospital adverse event nexus, which we established above.

**Table 5:** Likelihood of experiencing hospital and non-hospital harm, ordered probit

	**(1)**	**(2)**	**(3)**	**(4)**	**(5)**	**(6)**
Married	−0.00422	0.0628	0.09277	−0.0610	0.0303	0.0395
	(0.0638)	(0.0622)	(0.0575)	(0.0619)	(0.0530)	(0.0577)
Divorced	0.0179	0.00655	0.0526	−0.0189	−0.0159	0.0274
	(0.0544)	(0.0558)	(0.0548)	(0.0662)	(0.0590)	(0.0625)
Widow	0.0385	0.103	0.138	−0.0575	0.0192	0.0458
	(0.0774)	(0.0858)	(0.0898)	(0.0587)	(0.0522)	(0.0638)
Primary education	−0.183	−0.239***	0.138	−0.0575	0.0192	0.0458
	(0.142)	(0.0894)	(0.132)	(0.143)	(0.0958)	(0.130)
Secondary education	−0.228***	−0.258***	−0.171**	−0.255***	−0.313***	−0.198**
	(0.0875)	(0.0726)	(0.0826)	(0.0824)	(0.0673)	(0.0778)
University education	−0.180**	−0.0384	−0.179**	−0.252***	−0.194***	−0.246***
	(0.0715)	(0.0689)	(0.0732)	(0.0768)	(0.0690)	(0.0.0774)
Female	−0.161***	−0.187***	−0.179***	−0.113***	−0.133***	−0.131***
	(0.0301)	(0.0323)	(0.0307)	(0.0336)	(0.0336)	(0.0319)
Urban	−0.00288	0.00489	0.00433	−0.00810	−0.00817	−0.00217
	(0.571)	(0.0406)	(0.0519)	(0.0525)	(0.0397)	(0.0480)
Household size	−0.0155	0.0167	0.0134	−0.0157	0.0114	0.0135
	(0.0250)	(0.0158)	(0.0196)	(0.0189)	(0.0119)	(0.0141)
Poor	−0.478***	−0.314***	−0.411***	−0.428***	−0.267***	−0.354***
	(0.0567)	(0.0585)	(0.0547)	(0.0683)	(0.0683)	(0.0683)
Age	−0.0144**	−0.0182***	−0.0161***	−0.00621	−0.00730	−0.00709
	(0.00577)	(0.00609)	(0.00588)	(0.00468)	(0.00472)	(0.00484)
Age squared	0.000209***	0.000195***	0.000165***	0.000175***	0.000134***	0.000121**
	(0.0000535)	(0.0000565)	(0.0000527)	(0.0000497)	(0.0000478)	(0.0000490)
Asset index	0.196***	0.0110	0.0115	0.228***	0.0173	0.0160
	(0.0568)	(0.0270)	(0.0387)	(0.0562)	(0.0274)	(0.0380)
_cut1	−11.03***	−11.54***	−14.24***	−10.78***	−11.50***	9.530**
	(1.047)	(1.061)	(4.211)	(1.025)	(1.043)	(4.284)
_cut2	−2.619***	3.063***	−5.812	−2.445***	−3.122***	−1.182
	(0.211)	(0.148)	(3.971)	(0.157)	(0.127)	(4.062)
_cut3	−0.336*	−0.587***	3.469	−0.141	−0.656***	1.178
	(0.204)	(0.137)	(4.003)	(0.152)	(0.101)	(4.080)
_cut4	2.529***	2.455***	−0.540	2.875***	2.507***	4.247
	(0.211)	(0.155)	(4.102)	(0.129)	(0.118)	(4.082)
Log of GDP per capita (average 2008-2010)			−0.277			0.150
			(0.441)			(0.438)
Total health expenditure (as % of GDP, average 2008-2010)			−0.0279			−0.00589
			(0.0813)			(0.0742)
Gini, average 2008-2010			−0.0662***			−0.00589
			(0.0197)			(0.0207)
Log of number of doctors per 1000 people (average 2008-2010)			0.538			0.221
			(0.613)			(0.521)
Corruption perception index (average 2008-2010)			0.195**			0.144*
			(0.0991)			(0.0805)
N	25016	25016	25016	24832	24832	24832
Country dummies	No	Yes	No	No	Yes	No
adj. R-sq						
Pseudo R-sq	0.010	0.060	0.027	0.011	0.053	0.026

Standard errors in parentheses.

=”*p < 0.1, **p < 0.05, ***p < 0.01”.

We then repeated the exercise above for all individual types of adverse events – hospital infections, incorrect diagnosis, surgical errors, medication related errors, and medical device errors. The results of that analysis broadly confirm our findings and are available upon request.

In addition to the analysis we conducted two more robustness checks. The first one assessed the likelihood of a person suffering an adverse event contingent upon their assessment of the quality of healthcare. The storyline that emerges from this analysis confirms our main findings from above. The second robustness check was conducted in order to establish if preferences for access versus treatment increase the likelihood of being exposed to an adverse event. Our results did not find statistically significant evidence for this, whilst providing evidence for our conclusions from above. As in the previous case, the results of this analysis are available upon request^k^.

### Additional robustness checks

In addition, we conducted a few overall robustness checks. In the first one, we ran a probit analysis using the dichotomised perception of health care quality as a dependent variable and the individual socio-economic characteristics as independent variables and then summarised the fitted values on a country by country basis. Ultimately, we conducted a simple correlation exercise between the obtained fitted values and the macro-economic variables used in the analysis above. Our results are reported in Additional file 3: Panel 1. The analysis broadly confirms our findings vis-à-vis corruption and inequality.

We conducted similar robustness checks for the likelihood of adverse events variables (both for hospital and non-hospital-related harm) and the results of our analysis are presented in Additional file 3: Panels 2 and 3. As in the case above, our results broadly confirm our initial conclusions^l^.

Our additional robustness check includes running correlations of the perception of quality of health care and individual health care attributes (both obtained as fitted values from a probit analysis on individual socio-economic and demographic characteristics). The results are presented in Additional file 3: Panel 4. Here we find evidence for a strong correlation between preferences for well-trained doctors and cleanliness of health care facilities on the one hand, and perceived quality of the health care on the other.

### Interaction between quality of health care and likelihood of adverse events

Our final analysis incorporated the perception of health care quality and likelihood of occurrence of adverse events. In other words, in our last exercise we tried to ascertain whether people with a higher opinion of the health care system also believe that the likelihood of an adverse event occurring is lower. In conducting the analysis we used a similar approach to the one described above – we plotted the fitted values obtained from a probit analysis on socio-economic and demographic characteristics for both perception of health care quality and the likelihood of adverse events occurring. Our results are presented in Additional file 1: Charts 1, 2 and 3. This analysis undoubtedly confirms the link between the two variables, i.e. people with better perception of health care quality also believe that the likelihood of adverse events is smaller and vice versa, for respondents with a negative perception of health care quality, the perceived likelihood of adverse events increases.

## Discussion

The main aim of this paper is to examine the perception of quality of health care, the main determinants of health care quality and to examine the perception of the likelihood of being harmed by different adverse events (both hospital and non-hospital-related).

A starting assumption underpinning our analysis was that both individual and macro-level characteristics would determine respondents’ perceptions of health care quality as well as the likelihood of being harmed by specific adverse events. We find evidence that age and socio-economic status, and to some extent, gender, act as main determinants of individuals’ perception of health care quality. Moreover, we find robust evidence for a non-linear impact of age on both the perceived quality of health care and the perception of being harmed by an adverse event. In that respect, our research feeds into an emerging body of social sciences research that finds a U-curve relationship between overall satisfaction with services and age.

Second and most important, we find evidence that two macro-level variables play a significant role in determining the perception of health care quality and the likelihood of being harmed by hospital and non-hospital care. Our analysis provides evidence for a positive association between transparency and the quality of health care. In other words, in countries with higher public sector transparency and lower corruption, the general public seems to be more satisfied with the quality of health care services. More importantly, the perception of the likelihood of being harmed by medical errors increases in countries that are more unequal, which is suggestive of the possibility of inadequate access to health care, and especially to quality health care, for lower socio-economic groups.

As noted previously, there are limitations to our analysis that mainly stem from a lack of variables that could help us control for the individual level of utilisation of health care services and for subjective health care evaluation. There are, however, ways to deal with these shortcomings and we believe that we have addressed them. First, we use a macro-level aggregate proxy for health care utilisation (total healthcare expenditure as a percentage of GDP). In addition, while the survey does not contain a measure of subjective health assessment, given the strong correlation between age and subjective health assessment, we believe that age could be used as a proxy for health status.

## Conclusions

There is a possibility for our results to be distilled into policy actions. Improving transparency and fighting corruption across all echelons of the public sector (but especially in the health care sector) would help to improve peoples’ perception of the overall quality of health care. Moreover, improving access to health care, especially access for the poorest socio-economic groups would help to improve not only their perception of overall health care quality, but it would also boost their confidence that they are entitled to good health care, and more importantly, care that is safe and free from harm.

Finally, our results also point to the fact that there is no panacea for an instantaneous improvement in perceptions of health care quality. Indeed, our analysis suggests that the perceptions of health care quality and safety are tied to much more endemic problems, such as corruption, low transparency and income inequality. This also points to the impossibility of a successful reform that would only target the health care system, or, even less so, a certain sector of the health care system. Reform that is all-encompassing, that would target all the echelons of the public sector and that would also promote equitable and inclusive growth could improve Europe’s general public perception of health care quality and patients’ safety.

## Endnotes

^a^In May 2004, ten new countries became EU members: Cyprus, Czech Republic, Slovenia, Slovakia, Poland, Estonia, Latvia, Lithuania, Malta and Hungary. In 2007, Bulgaria and Romania also joined.

^b^For the purpose of this literature review, the term “patients” also comprises the general public that uses healthcare services.

^c^A detailed description of the variables used in the model is provided in Additional file 1: Table A2.

^d^Additional file 4: Table A3 provides a detailed account of the questions used as dependent variables.

^e^However, there are proxies that could be used in this instance, such as, for example, age.

^f^The conclusions that stem from this exercise closely correspond to our findings that rely on the usage of country dummies. The country dummies suggest a strong link between quality of care and countries that are more egalitarian and have a better institutional set up. The results of this exercise are available upon request.

^g^In addition, we employ a secondary robustness check, i.e. we experiment with using age as a categorical variable. The results that we obtain broadly correspond to our main findings. Furthermore, they are available upon request.

^h^In order to validate our results, we also conducted a secondary exercise, i.e. we ran our basic model on individual countries’ data. The results that emerge from that analysis confirm our main results vis-à-vis the independent variables – gender, age and income categories seem to matter when assessing the quality of healthcare. There are notable differences (both in terms of sign and significance across countries) which are picked up by country dummies in the cross-sectional part of the analysis.

^i^These results are available upon request.

^j^The results table does not report the coefficients of the individual nor macro-level variables, but they are available on request.

^k^Results are available upon request.

^l^In addition, we also conducted secondary robustness checks, whilst also using more lenient measures of quality as well as a more lenient measure of the perceived likelihood of being affected by adverse events. Even with these newly established variables, our results strictly follow our already established relationship between perception of quality and likelihood of adverse events and macro-economic variables.

## Abbreviations

EU: European union; GDP: Gross domestic product; HMO: Health maintenance organization; OECD: Organisation for economic cooperation and development; OLS: Ordinary least squares; PPP: Purchasing power parity; UK: United Kingdom.

## Competing interests

The authors declare that there are no competing interests.

## Authors’ contributions

Both authors were involved in the process of inception and drafting of the manuscript. EM provided the overall framework for the analysis, while ZN conducted the statistical analysis. ZN provided the write up for the background, methods and results part of the paper, while EM drafted the discussion and the conclusion. Both authors were involved in editing of the paper. Both authors read and approved the final manuscript.

## Pre-publication history

The pre-publication history for this paper can be accessed here:

http://www.biomedcentral.com/1472-6963/13/472/prepub

## Supplementary Material

Additional file 1: Table A2 Further definition of variables, availability and source. Click here for file

Additional file 2: Table A1 Perception of quality of healthcare: OLS and probit. Click here for file

Additional file 3 **Panel 1.** Perception of quality of healthcare and selected macro variables. **Panel 2**. Likelihoodof hospital harm and selected macro variables. **Panel 3**. Likelihoodof non-hospital harm and selected macro variables. **Panel 4**. Perception of quality of healthcare and attributes of healthcare. **Chart 1**. Perception of healthcare quality and adverse events. **Chart 2**. Perception of quality and healthcare system. **Chart 3**. Quality of healthcare system and adverse events. Click here for file

Additional file 4: Table A3 Questions used as dependent variables in the analysis. Click here for file
