# Supplementary material for: Corruption, inequality and population perception of healthcare quality in Europe
Source: BMC Health Serv Res. 2013 Nov 11;13:472. doi: 10.1186/1472-6963-13-472 (PMC3831823; doi:10.1186/1472-6963-13-472)
Supplement: Supplementary file 1 [file 1472-6963-13-472-S1.docx]

Table A2. Further definition of variables, availability and source

	proxy	years	source
GDP per capita, PPP (constant USD, 2005) average 2008-2010	usually used as a proxy for economic development. The regressions use log of the three year averages	2008-2010	WDI
Transparency International Corruption Perception Index	used as a proxy for corruption. Measured on a 1-10 scale (10 - best).	2008-2010	Transparency International
Gini, average 2008 - 2010	used as a proxy for inequality	2008-2010	Eurostat
Health care expenditure per capita, in USD, average 2008-2010	used as a proxy for monetary inputs in healthcare systems	2008-2010	WDI
Publich health expenditure (in % of GDP), average 2008-2010	used as a proxy for monetary inputs in healthcare systems	2008-2010	WDI
Health care expenditure (in % of GDP), average 2008-2010	used as a proxy for monetary inputs in healthcare systems	2008-2010	WDI
Nurses (per 1,000 people), average 2008-2010	used as a non-monetary input in healthcare	2008-2010	WDI
Physicians (per 1,000 people), average 2008-2010	used as a non-monetary input in healthcare	2008-2010	WDI
Unemployment (in %), average 2008-2010	proxy for overall welfare/wellbeing	2008-2010	WDI

Source: WDI, Eurostat, Transparency International
