# Supplementary material for: Corruption, inequality and population perception of healthcare quality in Europe
Source: BMC Health Serv Res. 2013 Nov 11;13:472. doi: 10.1186/1472-6963-13-472 (PMC3831823; doi:10.1186/1472-6963-13-472)
Supplement: Supplementary file 2 [file 1472-6963-13-472-S2.docx]

Table A1. Perception of quality of healthcare: OLS and probit

	OLS	OLS	OLS	probit	probit	probit
	(1)	(2)	(3)	(4)	(5)	(6)
married	0.0695**	-0.0224	-0.0220	-0.130***	0.0440	0.0354
	(0.0262)	(0.0144)	(0.0196)	(0.0451)	(0.0348)	(0.0392)
divorced	0.00673	-0.0149	-0.0315	-0.0801	-0.0362	-0.0108
	(0.0301)	(0.0209)	(0.0303)	(0.0518)	(0.0413)	(0.0568)
widow	0.0793**	-0.0242	-0.0218	-0.209***	-0.0234	-0.0351
	(0.0315)	(0.0214)	(0.0268)	(0.0526)	(0.0435)	(0.0508)
primary education	0.0190	0.0326	0.0482	-0.0184	-0.0536	-0.105
	(0.0540)	(0.0397)	(0.0451)	(0.0901)	(0.0704)	(0.0779)
secondary education	0.0859*	0.0650**	0.0398	-0.0995	-0.0930*	-0.0598
	(0.0438)	(0.0277)	(0.0409)	(0.0711)	(0.0498)	(0.0708)
university education	0.0153	0.0163	0.0198	0.00250	-0.0166	-0.0177
	(0.0394)	(0.0297)	(0.0334)	(0.0599)	(0.0548)	(0.0609)
female	0.00248	0.0213*	0.0192*	0.0316	0.00121	0.00534
	(0.0134)	(0.0110)	(0.0110)	(0.0203)	(0.0220)	(0.0201)
urban	0.0286	0.00696	0.0288	-0.0361	-0.0102	-0.0330
	(0.0291)	(0.0124)	(0.0202)	(0.0468)	(0.0274)	(0.0373)
household size	0.0239**	-0.00450	-0.00300	-0.0300*	0.0149*	0.0117
	(0.0107)	(0.00349)	(0.00638)	(0.0155)	(0.00813)	(0.0115)
poor	0.192***	0.117***	0.125***	-0.288***	-0.224***	-0.216***
	(0.0307)	(0.0266)	(0.0302)	(0.0564)	(0.0594)	(0.0622)
age	0.0117***	0.00959***	0.0106***	-0.0150***	-0.0150***	-0.0147***
	(0.00247)	(0.00211)	(0.00223)	(0.00440)	(0.00419)	(0.00433)
age squared	-0.000202**	-0.000118**	-0.000134**	0.000297***	0.000197***	0.000205***
	(0.0000247)	(0.0000206)	(0.0000232)	(0.0000459)	(0.0000404)	(0.0000428)
asset index	-0.228***	-0.0122	-0.0249**	0.361***	0.0166	0.0385*
	(0.0300)	(0.00867)	(0.0115)	(0.0501)	(0.0203)	(0.0222)
Log of GDP per capita (average 2008-2010)			-0.275			0.407
			(0.190)			(0.396)
Health expenditure (as % of GDP, average 2008-2010)			-0.00614			0.00977
			(0.0313)			(0.0671)
Gini (average 2008-2010)			0.0176			-0.0310
			(0.0154)			(0.0265)
Log of number of doctors per 1000 people (average 2008-2010)			-0.0656			0.0896
			(0.261)			(0.424)
Corruption Perception Index (average 2008-2010)			-0.126***			0.237***
			(0.0360)			(0.0585)
_cons	2.051***	1.730***	5.277**	0.639***	1.321***	-4.204
	(0.104)	(0.0474)	(2.060)	(0.177)	(0.109)	(4.074)
N	25661	25661	25661	26025	26025	26025
R-sq	0.086	0.314	0.234			
adj. R-sq	0.086	0.313	0.233			
pseudo R-sq				0.061	0.242	0.179
Country dummies	NO	YES	NO	NO	YES	NO

Standard errors in parentheses
="* p<0.1 ** p<0.05 *** p<0.01"
