# Supplementary material for: Corruption, inequality and population perception of healthcare quality in Europe
Source: BMC Health Serv Res. 2013 Nov 11;13:472. doi: 10.1186/1472-6963-13-472 (PMC3831823; doi:10.1186/1472-6963-13-472)
Supplement: Supplementary file 3 [file 1472-6963-13-472-S3.docx]

Panel 1. Perception of quality of healthcare and selected macro variables

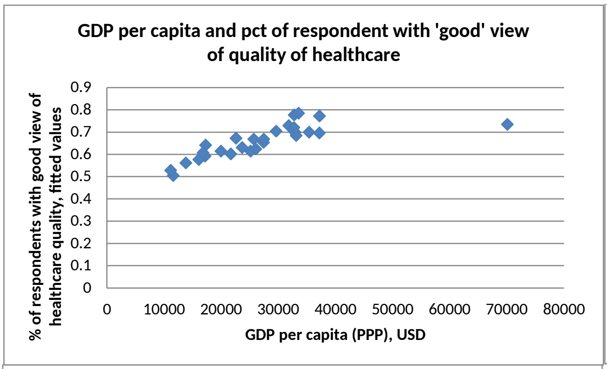

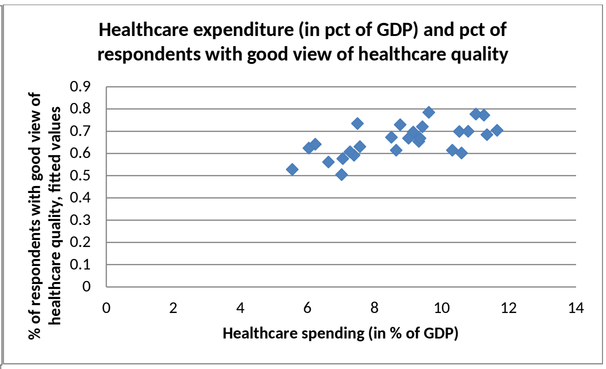

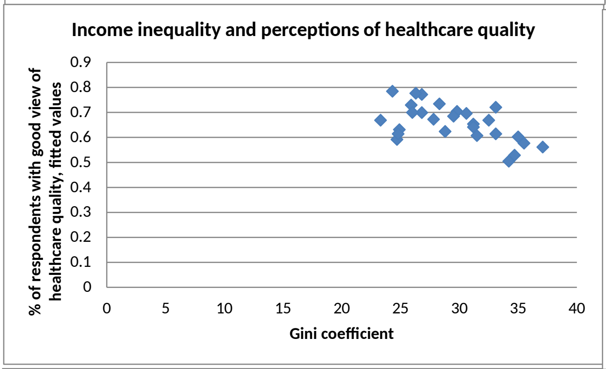

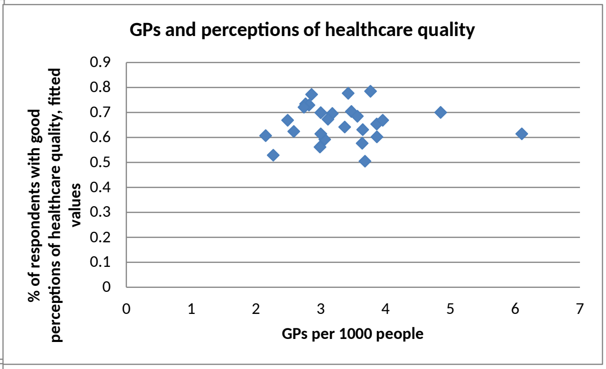

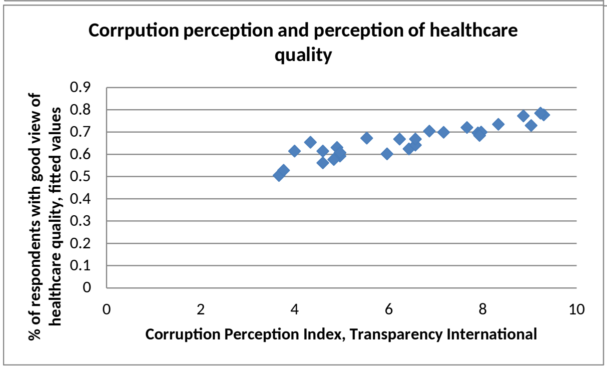

Panel 2. Likelihood of hospital harm and selected macro variables

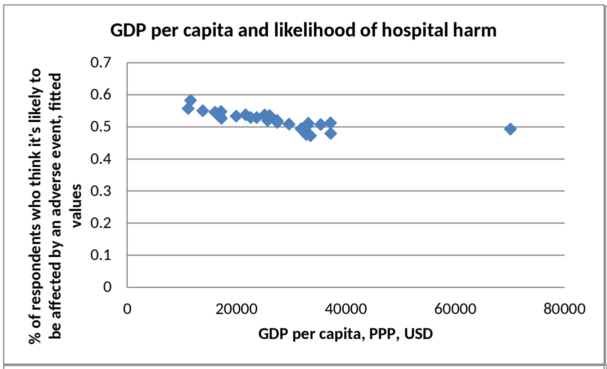

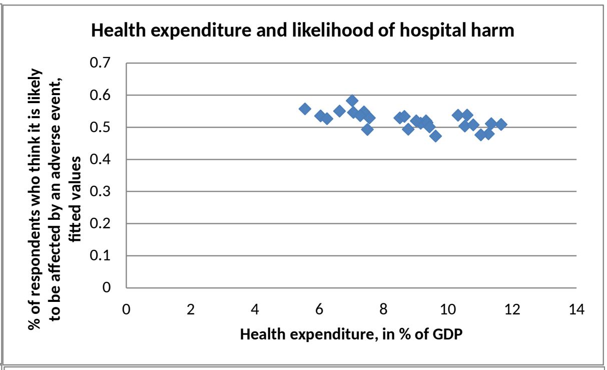

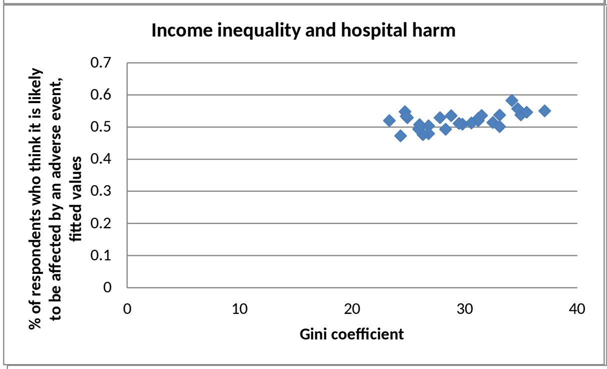

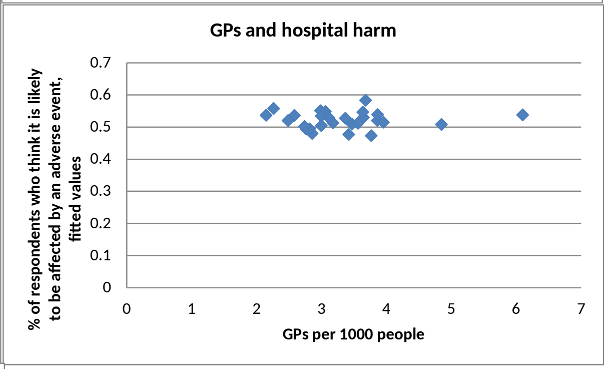

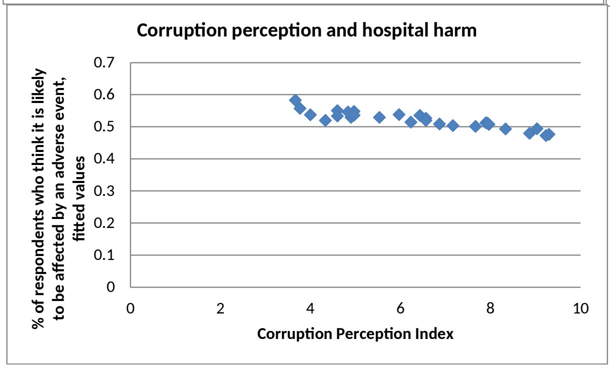

Panel 3. Likelihood of non-hospital harm and selected macro variables

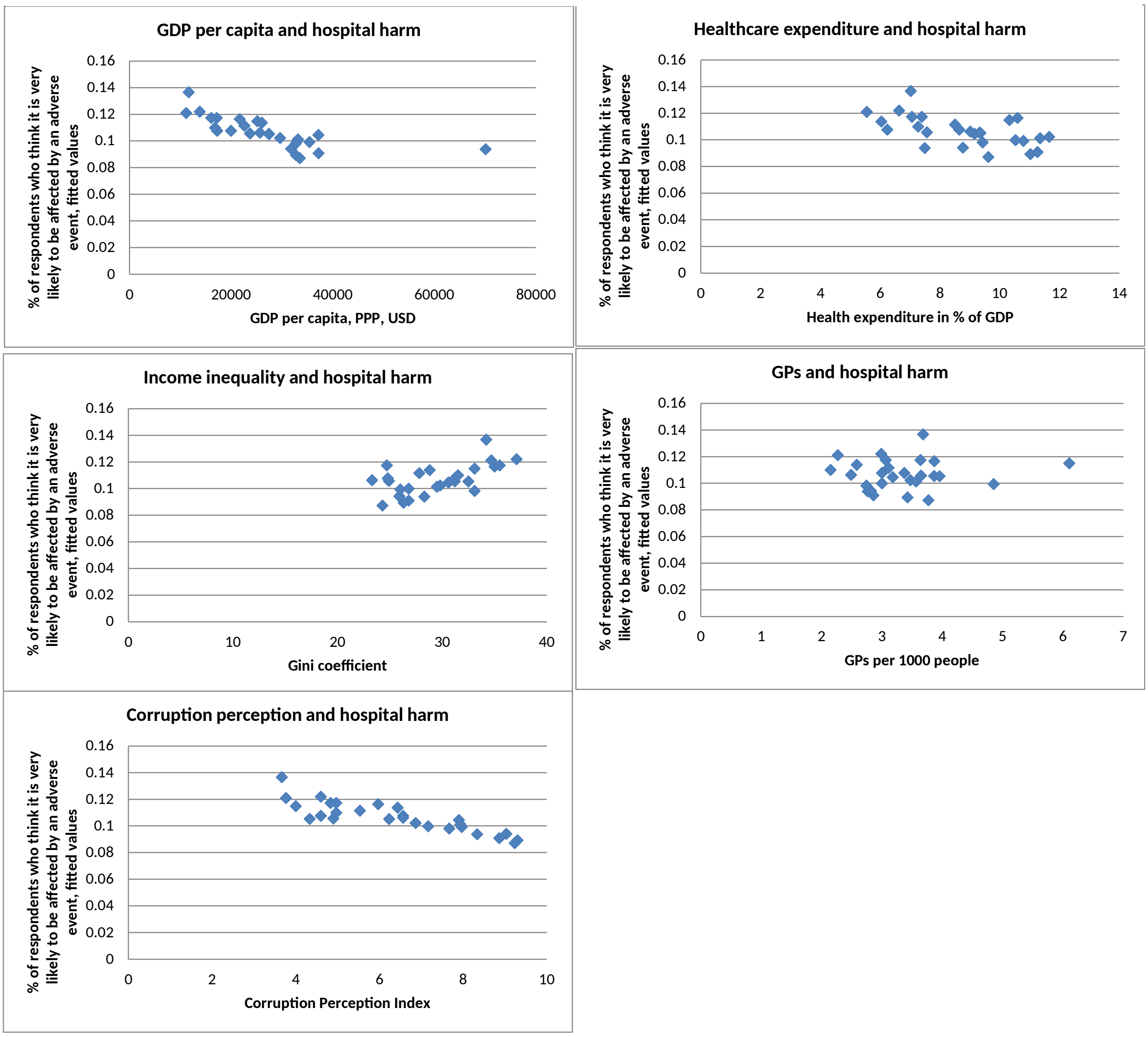

Panel 4. Perception of quality of healthcare and attributes of healthcare

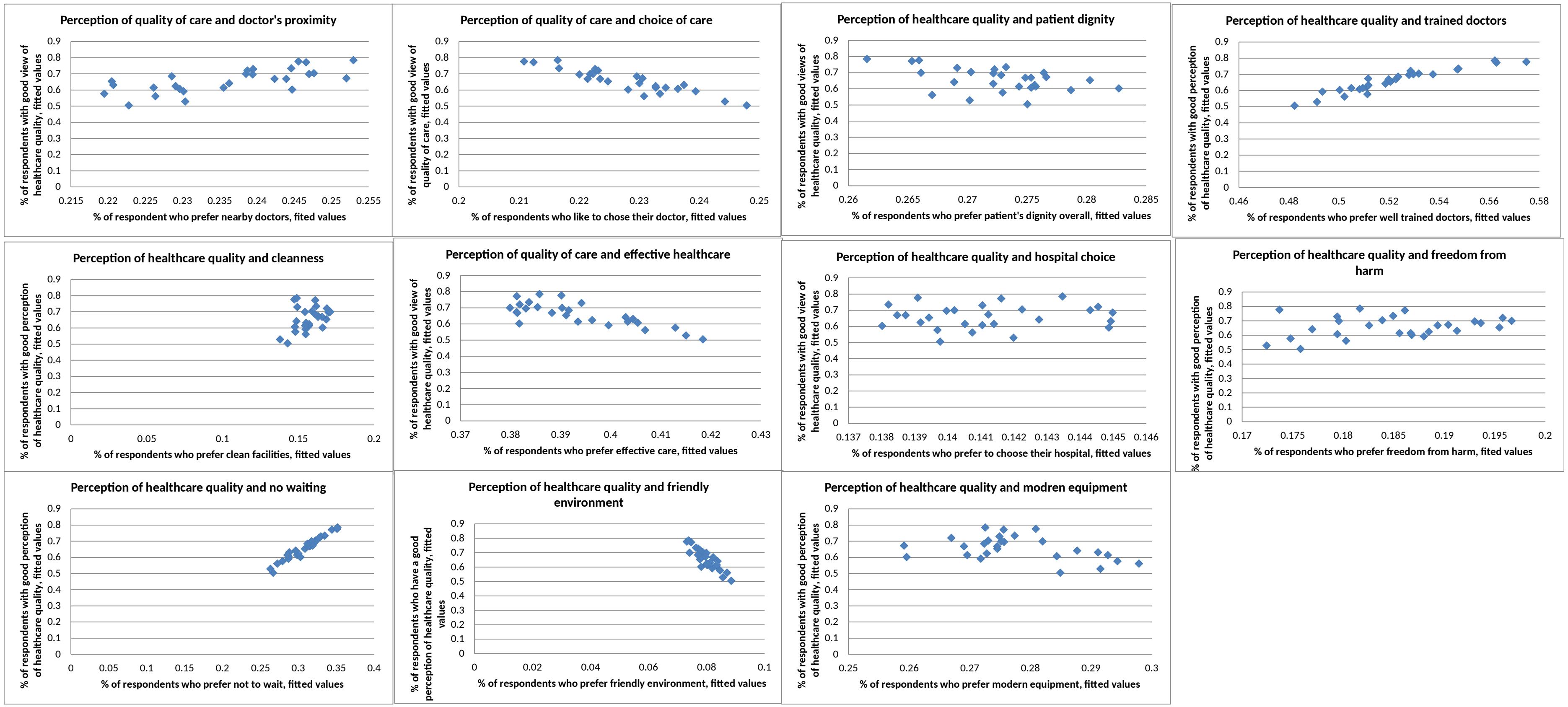

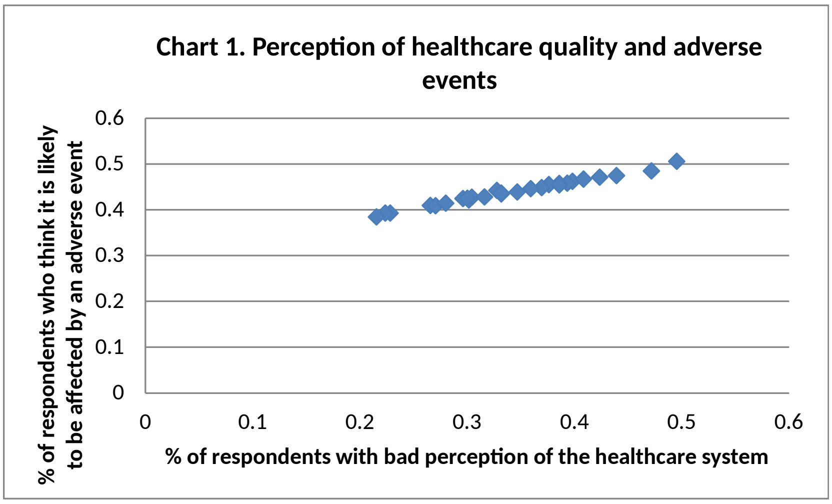

Chart 1. Perception of healthcare quality and adverse events
% of respondents who think it is likely to be affected by an adverse event
0.6
0.5
0.4
0.3
0.2
0.1
0
0 0.1 0.2 0.3 0.4 0.5 0.6
% of respondents with bad perception of the healthcare system

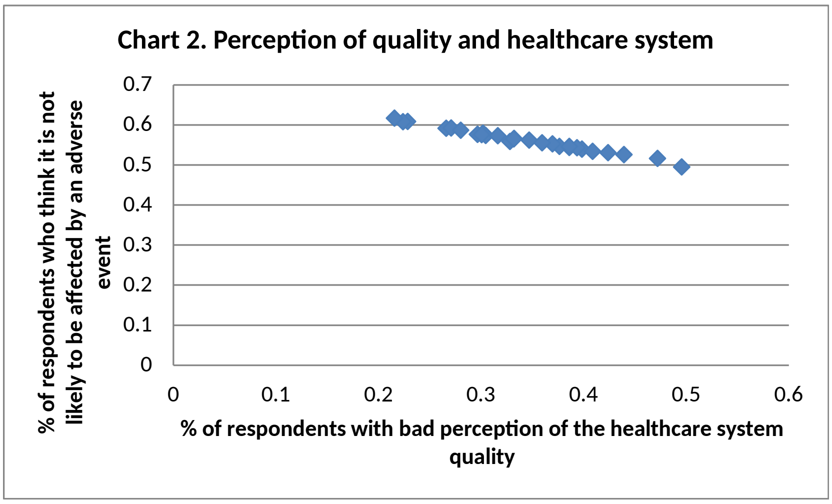

Chart 2. Perception of quality and healthcare system
% of respondents who think it is not likely to be affected by an adverse event
0.7
0.6
0.5
0.4
0.3
0.2
0.1
0
0 0.1 0.2 0.3 0.4 0.5 0.6
% of respondents with bad perception of the healthcare system quality

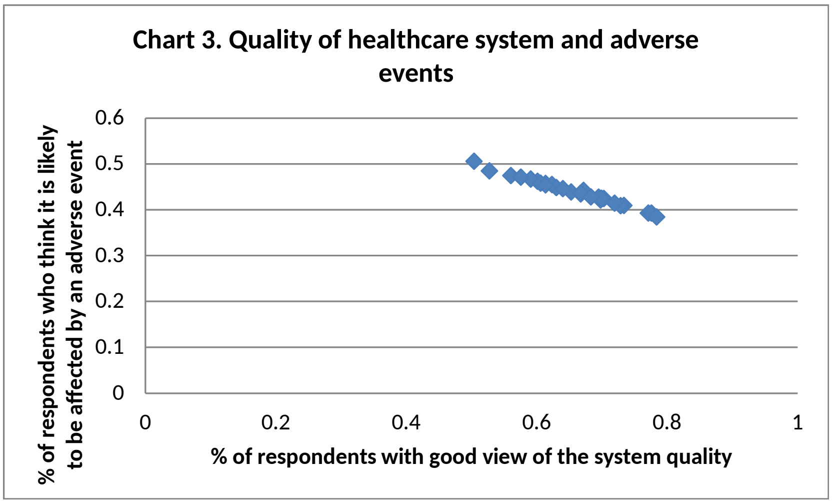

Chart 3. Quality of healthcare system and adverse events
% of respondents who think it is likely to be affected by an adverse event
0.6
0.5
0.4
0.3
0.2
0.1
0
0 0.2 0.4 0.6 0.8 1
% of respondents with good view of the system quality
