# Supplementary material for: Corruption, inequality and population perception of healthcare quality in Europe
Source: BMC Health Serv Res. 2013 Nov 11;13:472. doi: 10.1186/1472-6963-13-472 (PMC3831823; doi:10.1186/1472-6963-13-472)
Supplement: Supplementary file 4 [file 1472-6963-13-472-S4.docx]

Table A3. Questions used as dependent variables in the analysis

QD1. Of the following criteria, which are the three most important criteria when you think of high quality healthcare in (OUR COUNTRY)?

Proximity of hospital and doctor
Free choice of doctor
Respect of a patient's dignity
Medical staff that is well trained
A clean environment at the healthcare facility
Treatment that works
Free choice of hospital
Healthcare that keeps you safe from harm
No waiting lists to get seen and treated
A welcoming and friendly environment
Modern medical equiptment

QD2. How would you evaluate the overall quality of healthcare in (OUR COUNTRY)?

Very good
Fairly good
Fairly bad
Very bad

QD4a. How likely do you think it is that pateints could be harmed by hospital care in (OUR COUNTRY)? By hospital care we mean being reated in a hospital as an outpatient or inpatient.

Verylikely
Fairly likely
Not very likely
Not at all likely

QD4b. And how likely do you think it is that patients could be harmed by non-hospital care in (OUR COUNTRY)? By non-hospital care we mean receiving diagnosis, treatment or medicine in a clinic or surgery of your general practitioner or from a farmacy.

Very likely
Fairly likely
Not very likely
Not at all likely

QD5. Being harmed when receiving healthcare is also referred to as "adverse events". Thinking of the following types of adverse events in your view, how likely, if at all, is it that each of these might happen to you if you were to receive healthcar ein (OUR COUNTRY)?

	Very likely	Fairly likely	Not very likely	Not at all likely
Hospital infections	1	2	3	4
Incorrect, missed or delayed diagnoses	1	2	3	4
Surgical errors	1	2	3	4
Medication related errors (wrong prescription, worng dose, dispensing error in pharmacy, wrong administration route)	1	2	3	4
Medical device or equipment related errors	1	2	3	4
